# Recent Advancement in Atypical Lipomatous Tumor Research

**DOI:** 10.3390/ijms22030994

**Published:** 2021-01-20

**Authors:** Emi Mashima, Yu Sawada, Motonobu Nakamura

**Affiliations:** Department of Dermatology, University of Occupational and Environmental Health, 1-1, Iseigaoka, Yahatanishi-Ku, Kitakyushu, Fukuoka 807-8555, Japan; e-mashima@med.uoeh-u.ac.jp (E.M.); motonaka@med.uoeh-u.ac.jp (M.N.)

**Keywords:** atypical lipomatous tumor, clinical characteristics, MRI, treatment, superficial type, deep type

## Abstract

After Evans and colleagues identified the lipomatous tumor with a well-differentiated liposarcoma in a subcutaneous location or within a muscle layer, namely, atypical lipomatous tumor (ALT), this malignancy has been investigated to clarify the characteristics of clinical behavior and genomic changes. As one of the important issues for clinicians, it is a hot topic of how to distinguish ALT from benign lipoma in the clinical aspect. Recent studies revealed novel findings to clarify the risk factor for the diagnosis of ALT and molecular targets for the treatment of ALT. Clinical characteristics of superficial-type ALT well reflect the subcutaneous location of the tumor and are slightly different compared to deep-type ALT, such as tumor size. In addition, there has been a recent discovery of novel findings in ALT-related genes, namely, HMG2A (high mobility group protein 2a), YEATS4 (YEATS domain containing 4), and CPM (Carboxypeptidase M). Recent updates on treatment for advanced ALT are well developed including immunotherapy and conducting clinical trials. Finally, this review introduces one of the hot topics of ALT research focused on epigenetic changes: their attention in recent updates on clinical characteristics and the novel discovery of related genes, treatment, and epigenetic modifications in atypical lipomatous tumors.

## 1. Introduction

Skin is located mostly on the outside of the human body and consists of three major layers: the epidermis, dermis, and subcutaneous layers. Among them, subcutaneous fat is the major component in the subcutaneous layer and is mainly composed of adipocytes. Traditionally, subcutaneous fat plays a major role in energy reserve, thermoregulation, and relief of the pressure from outside of the human body [1,2]. In addition, recent studies identified that the fat layer plays an additive function such as antimicrobial actions [3]. These findings provide us with a more detailed, precise, important role of the subcutaneous layer in the human body. Therefore, subcutaneous layer-mediated human diseases are recently highlighted issues for clinicians.

Adipocyte-derived tumors are commonly observed in various ages and locations in the skin. A representative tumor is known as a lipoma, which is commonly observed in ordinal clinics. However, sometimes, dermatologists encounter the malignant form of the adipocyte-derived tumor, namely, liposarcoma, which exhibits an unfavorable clinical outcome with a higher frequency of the local recurrence rate and the advanced form is intractable by the current systemic treatment. Among liposarcomas, the atypical lipomatous tumor (ALT) is a major form of liposarcomas. ALT is a malignancy derived from adipocytes and is synonymous with a well-differentiated liposarcoma, which occupies 46% of all liposarcomas [4]. Evans and colleagues identified the lipomatous tumor as a well-differentiated liposarcoma in a subcutaneous layer or within a muscle layer [5] with higher frequencies of the local recurrence rate and distant organ metastasis. They named this tumor with unique clinical characteristics as ALT. Based on previous studies, ALT is divided into two clinical subtypes: the superficial type located in a fat layer origin, and the deep type located in a retroperitoneum or muscle layer origin [6]. The superficial type is a rare subtype of ALT [7,8,9,10,11,12,13,14,15,16,17,18,19,20,21,22], and little is known about the clinical characteristics of superficial-type ALT [23]. To clarify this issue, our group recently reported the detailed clinical characteristics of superficial-type ALT.

In this review, we focused on the recent hot topics of clinical characteristics of ALT, in addition to the recent discovery of ALT-related genes, treatment, and epigenetic modification in ALT. 

## 2. Risk Factor

There have been several reported clinical parameters as the Merck marks for the diagnosis of ALT. Age more than 60 years old, the size of the tumor more than 10 cm, and a tumor location in the thigh are recognized as indicators for the diagnosis of ALT [24]. Male sex was reported as a risk factor for the diagnosis of ALT by Kransdorf et al. [25].

Our recent study identified that superficial-type ALT shows slightly different clinical manifestations compared with deep-type ALT [23]. In estimating tumor invasion and adhesion of the tumor, tumor mobility is considered poorer in superficial-type ALT and superficial-type ALT is considered to exhibit a hard texture. The tumor size in superficial-type ALT becomes bigger than ordinal lipoma and shows a tumor size of more than 4 cm. In particular, tumor size in superficial-type ALT seems to be smaller compared with that in deep-type ALT. This information does not represent the different characteristics of the tumor between deep-type and superficial-type ALT. However, it is assumed that superficial-type ALT is easily noticed by patients themselves as a subcutaneous mass in the early stages. In addition, superficial-type ALT exhibits a higher frequency of the tumor site at extremities (40%) compared to benign lipoma (12.7%). The frequency of ALT in the trunk is lower than benign lipoma, while superficial-type ALT is highly observed in the shoulders and buttocks.

Lipomatous tumors in pediatric patients are rather rare and a recent histopathological study on lipomatous tumors in 50 pediatric patients in a single institution showed 26 cases of lipoma (52%), 19 cases of lipoblastoma (38%), 3 cases of liposarcoma (6%), and 2 cases of ALT (4%) [26]. These findings prompted us to investigate more detailed characteristics of ALT in the pediatric population.

## 3. Imaging Examination

### 3.1. MRI

Many studies have conducted an MRI examination to clarify the characteristics of ALT. The signal intensity is equal between lipoma and ALT. The tumor margins of ALT and benign lipoma are well defined and smooth in almost all cases. Gaskin et al. analyzed MRI in patients with an adipocyte-derived tumor, and ALT showed low intensity in T1-weighted MRI and high intensity in T2-weighted MRI with a bulkhead structure and septal structures more than 2 mm in diameter with a sensitivity of 100% and specificity of 86% [27]. Another study also reported that 85.1% of ALT patients exhibited septal structures of more than 2 mm in thickness [28]. The septal structure in well-differentiated liposarcomas is enhanced on fat-suppressed T1-weighted MRI after contrast enhancement media administration [29]. Superficial-type ALT also exhibits similar characteristics in MRI [23].

A report indicated that contrast enhancement sometimes causes misleading of the findings of ALT and benign lipoma. Deep and proximal lower limb lesions show inadequate fat suppression and architecture findings in ALT cases without the contrast enhancement. On the other hand, MRI with contrast enhancement changes the impression of diagnosis incorrectly changed from benign lipoma to ALT [30]. Therefore, these findings suggest to us the importance of the combination of investigation including physical examination and imaging for the diagnosis of ALT.

### 3.2. 18F-FDG PET/CT

18F-FDG is commonly used for the assessment of the degree of metabolism of glucose to evaluate various organs and the detection of tumors in oncology fields. 18F-FDG PET scanning enables us to evaluate 18F-FDG uptake in the tumor by the standardized uptake value (SUV), which reflects rapidly growing malignant tumors [31]. SUVmax is well reflected in the case of ALT and the dedifferentiated form as a marker of the advancement of lipomatous tumors [31].

## 4. ALT-Associated Genes

In ALT, high positive amplification for *MDM2* (murine double minute 2) and *CDK4* (cyclin-dependent kinase 4) within 12q13-15 is frequently observed [32]. ALT also shows other gene amplifications within 12q13-15, including *HMG2A* (high mobility group protein 2a), *YEATS4* (YEATS domain containing 4), and *CPM* (Carboxypeptidase M). *HMG2A* encodes a transcriptional factor for cellular transformation [32]. *YEATS4* and *CPM* are known to be associated with the dedifferentiation of liposarcoma. *YEATS4* is essential for the suppression of a tumor suppressor gene, *TP53*, and indeed its silencing-dedifferentiated liposarcoma tumor cells impair its proliferation [33]. *CPM* is responsible for carboxypeptidase M production which is a cleavage enzyme for growth factor and *CPM*-silencing tumor cells show reduced tumor growth and invasion [34]. 

Dedifferentiated liposarcoma also exhibited recurrent amplification within 1p32 and 6q23, which are associated with unfavorable clinical behavior. *JUN* (Jun proto-oncogene) and *ASK* (apoptosis signal-regulating kinase) are located in 1p32 and 6q23, respectively, and inhibit adipocyte differentiation mediated by peroxisome proliferator-activated receptor-gamma (PPARγ) [35]. Receptor tyrosine kinase (RTK) genes *DDR2* (discoidin domain receptor tyrosine kinase 2), *ERBB3* (Erb-B2 receptor tyrosine kinase 3), *FGFR1* (fibroblast growth factor receptor 1), and *ROS1* (ROS Proto-Oncogene 1) are also amplified in dedifferentiated liposarcoma [36]. Other genes such as *RB1* (Retinoblastoma 1), *ATM* (Ataxia telangiectasia mutated), *CHEK1* (Checkpoint kinase 1), RUNX3 (*RUNX family transcription factor 3*), and *ARIDIA* (Adenine thymine-rich interactive domain 1A) are also identified as genes associated with unfavorable clinical behavior [32]. 

## 5. Histological Analysis

Histological analysis is helpful for the diagnosis of ALT with the existence of atypical spindle or pleomorphic stromal cells within thickened fibrous septa, associated with multivacuolated lipoblasts [23].

As an aid for the diagnosis of ALT, amplification of the chromosome 12q13-15 region is confirmed in ALT, containing the *MDM2* gene and the *CDK4* gene [37] by FISH staining. Since every institution and hospital cannot necessarily conduct FISH staining, several types of research evaluate the utility of immunostainings of MDM2 and CKD4 (Table 1). Previous studies have already shown the frequencies of these positivities in ALT by immunostaining and these specificities of MDM2 and CDK4 were 82.8–100% (total mean 94.7%) and 69.0–100% (total mean 88.8%). Clay et al. analyzed the immunohistochemical staining of MDM2 and CDK4 in 56 cases of ALT. In ALT, MDM2 exhibited 45% sensitivity and 98% specificity, and CDK4 exhibited 41% sensitivity and 92% specificity. Furthermore, staining was observed exclusively in atypical cells, not in adipocytes, indicating that it might be a useful diagnostic tool for ALT [38]. 

As other aids for the diagnosis, Tenascin C and Cathepsin D expressions are upregulated in ALT than in lipomas [39]. Tenascin C is a glycoprotein component of the extracellular matrix and cathepsin D is an aspartyl lysosomal protease. Both contribute to the development of the tumor, invasion, and metastasis. YKL-40 is a glycoprotein expressed in various malignant tumors. YKL-40 expression increases in liposarcoma and is associated with poor prognosis [40]. Histologically, both CDK4 and JUN upregulations were observed in dedifferentiated liposarcoma, whereas amplification of HMGA2 was associated with the non-dedifferentiated form of ALT. The poor recurrence rate is associated with CDK4 and JUN upregulation. Recurrence-free survival is associated with HMGA2 [41]. ALT showed amplification of *MDM2* and carboxypeptidase M by fluorescence in FISH [42]. Upregulation of *DDIT3* (DNA damage inducible transcript 3) was observed in 33% of dedifferentiated liposarcoma patients and these patients were younger compared to those without *DDIT3* amplification [43].

There are several studies which evaluated the utility of p16 in ALT. p16 is an important cell cycle regulator. The expression of p16 was detected in 83.3% of ALT by immunohistochemical staining, while none of the lipomas were positive for p16 [44]. On the other hand, it seems there are some limitations in using p16 as a tool for the diagnosis of ALT due to the low specificity. Other studies showed the sensitivity and specificity in p16 were 89.5–100% and 60–92% by immunohistostaining [45,46,47]. Although single p16 analysis by immunostaining is limited to the utility, the combination with MDM2 and CDK4 immunostaining might be helpful for the diagnosis of ALT and dedifferentiated liposarcoma [47].

## 6. A Recent Update on the Prognosis of ALT

Regarding the local recurrence rate of ALT, it was reported that 91% of patients with retroperitoneal disease showed a high local recurrence and unfavorable clinical behavior [4]. On the other hand, the local recurrence rate of ALT in the muscle layer in extremities was 43% [4]. Therefore, the principle of treatment is an expanded surgical resection for deep-type ALT because repeating local recurrence might increase the risk of dedifferentiation of the tumor [53]. 

A systematic review analysis by using 20 studies revealed that there is no metastatic disease in ALT patients [54], and the local recurrence rate in patients with ALT was 15.1%. The average time to recurrence was 39 months from the date of surgical resection. In this analysis, 93.7% of patients with ALT were marginally resected and the overall recurrence rate was 15.1% after the surgical resection. The local recurrence rate after a wide resection was lower than marginal resection and was observed in 3.3% of ALT patients [24]. Another report indicated that ALT arising in the extremities had lower recurrence rates than deep-type ALT with 5-year recurrence-free survival of 88.9% and 59.0%, respectively, while patients with deep-type dedifferentiated liposarcoma show a greater unfavorable clinical course with 5-year event-free survival of 11.9% [55]. The actual reason remains unclear why deeper-type ALT tends to have an unfavorable clinical behavior, though it might be easy to access or recognize the presence of a superficial tumor because the tumor size in superficial-type ALT seems to be small compared with deep-type ALT [23]. That means superficial-type ALT might be recognized at an early stage and patients will get to start their treatment.

Dedifferentiation is observed in deep-type ALT, especially of a retroperitoneal origin; however, dedifferentiation is rare in subcutaneous tumors [29]. Previously published case reports of superficial-type ALT showed 16 cases of superficial-type ALT [7,8,9,10,11,12,13,14,15,16,17,18,19,20,21,22] with no local recurrence, showing a possible favorable prognosis of superficial-type ALT (Table 2). The difference in the prognosis may also reflect the difficulty in recognizing the tumors in a deep type of ALT, which leads to incomplete tumor resection. The cases of superficial-type ALT are easier to perform complete resection on and to recognize the tumors in an early stage, which results in a lower local recurrence after the tumor resection even with a marginal resection. However, clinicians keep in mind that there is no clear evidence for a prognosis difference between deep-type and superficial-type ALT and the standard therapeutic approach should be selected in the treatment of ALT, even in superficial-type ALT.

## 7. Treatment

### 7.1. Local Tumor Treatment

The consensus has been obtained that wide excision is the standard treatment in localized liposarcoma. Deep-type ALT becomes a candidate for the treatment for adjuvant or neoadjuvant radiotherapy [32]. Due to the difficulty in determining the perioperative management, especially regarding retroperitoneal ALT, the treatment should be determined in each case.

### 7.2. Systemic Treatment

In this section, we summarize the update on the current treatment of the advanced form of ALT.

#### 7.2.1. Anthracycline-Based Treatment

In advanced disease, anthracycline-based treatment is the first-line standard systemic treatment in the case of unresectable and metastatic cases [32]. However, an anthracycline-based regimen, such as an anthracycline single agent or anthracycline with the combination of the alkylating agent ifosfamide, has not necessarily led to a satisfactory clinical outcome. Several recent randomized phase 3 trials could not obtain an improvement in overall survival with the addition of ifosfamide or other types of alkylator agents plus doxorubicin in soft tissue sarcoma metastasis patients [56,57,58]. A combination of ifosfamide plus doxorubicin therapy improved the median progression-free survival from 4.6 to 7.4 months and the response rate from 14 to 26% compared to the doxorubicin single agent; however, there was no significant difference in the overall survival rate [56]. Therefore, among the limited number of therapeutic options, both the anthracycline alone agent or the combination anthracycline therapy obtained consensus as to the first-line treatment against advanced dedifferentiated liposarcoma.

#### 7.2.2. Eribulin

Eribulin in randomized phase 3 trial showed improved overall survival in advanced liposarcoma patients compared with dacarbazine [59]. Eribulin interferes with the prolonged mitotic inhibition and tumor cell apoptosis by suppression of mitotic spindle formation and cell cycle arrest during the G2–M phase to cause apoptosis [60]. Eribulin showed improved overall survival at 13.5 months compared with 11.5 months of being treated with dacarbazine [59]. In addition, another study revealed that dedifferentiated liposarcoma patients treated with eribulin exhibited 18.0 months overall survival compared with 8.1 months received with dacarbazine. On the other hand, no significant difference was observed in the median progression-free survival [61]. Consistently, dedifferentiated liposarcoma with doxorubicin resistance in a patient-derived retroperitoneal liposarcoma xenograft model revealed that eribulin inhibited dedifferentiated liposarcoma tumor development without any antitumor effect in the docetaxel treatment [62].

#### 7.2.3. Pazopanib

The multitarget tyrosine kinase inhibitor pazopanib also showed efficacy against liposarcoma. A phase 2 study revealed the following: a progression-free rate at 12 months of 68.3%; at 24 months, 39% of patients remained progression-free; and 44% of patients experienced tumor control. The median progression-free survival and the median overall survival were 4.4 and 12.6 months, respectively [63]. Another phase 2 study showed that out of all patients who received pazopanib at 800 mg per day, progression-free survival at 12 weeks was achieved in 43.2% of patients. The median progression-free survival and overall survival were 3.5 and 16.4 months, respectively. [64]. In a randomized clinical trial assigned to doxorubicin or pazopanib, pazopanib exhibited no inferior results in a similar quality of life measure outcome observed in doxorubicin treatment without hematological toxicity in pazopanib [65], which means pazopanib is possibly a favorable clinical option for elderly patients with dedifferentiated liposarcoma. Patient-derived dedifferentiated liposarcoma xenograft models showed that pazopanib inhibits tumor development by suppression of proliferation and angiogenesis [66].

#### 7.2.4. Trabectedin

Trabectedin is applicable to advanced liposarcoma, showing a progression-free survival rate compared with dacarbazine treatment [67]. Trabectedin exhibits a unique chemical structure with three fused tetrahydroisoquinoline rings. Among these rings, subunits A and B interact with the minor groove of the double helix in DNA. In addition, the subunit C provides a protrusion form of the DNA structure, which is enabled to interact with nuclear proteins [68]. These chemical component interactions allow driving interference with DNA binding proteins and transcription factors. In addition, trabectedin regulates cytokine and chemokine production by tumor and host cells to drive antitumor immunity [68]. Trabectedin suppresses adipocytic differentiation and tumor growth [69].

### 7.3. Development of Novel Treatment

#### 7.3.1. MDM2-Targeted Therapy

MDM2 is a negative regulator for the *p53* gene, which protects cells against malignant transformation [70]. MDM2 amplification is observed in ALT and its dedifferentiated form and even in other various sarcomas and carcinomas [71]. Therefore, blockage of the interaction between MDM2 and p53 has been thought of as an antitumor therapeutic option for liposarcoma. A phase 1 trial of SAR405838, which is an inhibitor of MDM2, showed no objective responses; however, a stable disease condition was observed in 65% of patients and a 3-month progression-free rate was observed in 32% of patients [72]. MK-8242 is also an inhibitor of MDM2 and shows partial responses in 7% of patients with liposarcoma and stable disease in 74% of patients [73]. One of the difficulties in the treatment with the MDM2 inhibitor is that *TP53* mutations appear in patients with dedifferentiated liposarcoma [74].

#### 7.3.2. CDK4-Targeted Therapy

CDK4 is one of the cell cycle regulators and the main action of CDK4-targeted therapy aims to suppress the phosphorylation of the RB protein and induce cell cycle arrest. Palbociclib is one of the inhibitors in both CDK4 and CDK6 and arrests the cell cycle in liposarcoma tumor cells [33]. In a clinical study, out of 48 patients with a well-differentiated liposarcoma and/or dedifferentiated liposarcoma treated with 200 mg, a 12-week progression-free rate was achieved in 66% of patients, and the 125 mg dose treatment achieved that in 57% of patients [75]. Another CDK4/6 inhibitor, ribociclib, showed that no objective responses were observed with stable disease in 15% of liposarcomas for 6 months [76].

#### 7.3.3. Exportin 1 Inhibitors

Exportin 1 exerts effects on the nuclear export of proteins associated with the development of liposarcoma progression and is upregulated in malignant tumors [77,78]. Tumor cell apoptosis and tumor growth inhibition are enhanced by silencing export 1 in liposarcoma cells [79]. In a clinical trial, an inhibitor of exportin 1, selinexor, showed no objective response; however, stable disease was observed in 47% of patients for 4 months without any achievement of partial response.

#### 7.3.4. PPARγ Agonists

PPARγ is a nuclear receptor that regulates gene transcription in liposarcoma and is essential for terminal adipocyte differentiation [80]. Consistently, PPARγ agonists enhance liposarcoma cell differentiation [81]. Sustained partial response was observed in a patient with myxoid liposarcoma [82].

#### 7.3.5. PD1/PDL1-Targeted Therapy

Recent advancements in immunotherapy against malignant tumors provide us novel therapeutic options in advanced-stage patients. In particular, programmed cell death protein 1 (PD1) acts as the suppression of immune responses and the enhancement of tolerance by the regulation of T cell-mediated immune activity. Programmed cell death ligand 1 (PDL1) is a ligand against PD1 and is a co-inhibitory factor of the immune response to reduce inflammatory cytokine secretion and cytotoxic reactions. The PD-1/PD-L1-mediated immune reaction is responsible for the immune escape phenomenon in malignant tumors. Therefore, this pathway-targeted immunotherapy is currently a hot topic for clinicians and will be desired for the treatment of advanced-stage liposarcoma.

A study showed liposarcoma patients exhibit high expression (92–100%) of PDL1 in well-differentiated liposarcoma tumor cells [83,84], and multivariate analysis revealed a PDL1-highly expressed sarcoma was closely associated with shorter survival [85]. In the dedifferentiated liposarcoma, high PDL1 expression was associated with statistically significant unfavorable recurrence-free survival and overall survival rates [86,87].

A phase II study of pembrolizumab in patients with advanced sarcoma showed favorable responses were observed in dedifferentiated liposarcoma [88,89]. Patients who respond to pembrolizumab tend to exhibit high infiltration of activated T cells and tumor-associated macrophages with PDL1 expression [89]. Highly infiltrated cytotoxic T cells also showed favorable progression-free survival [89].

A novel trial to enhance immune checkpoint inhibitor monotherapy has been developed by using an oncolytic vaccinia virus (GLV-1h68) to enhance the antitumor immune reaction and promote the response to PD-1 blockade in liposarcoma. Pretreatment with GLV-1h68 improves responses to the PD-1 inhibitor with an enhancement of CD8^+^ T cells infiltration and activation of dendritic cells [90].

#### 7.3.6. Aurora Kinases Inhibitor

The Aurora kinases are a serine/threonine kinases family, which plays an essential role in the cell cycle during M and G2 phases. Aurora A/B organize to regulate chromosome alignment [91]. Aurora A is essential for the management of genomic integrity [92]. Overexpression has been recognized in human tumors and correlates with tumor proliferation rates [93,94]. Alisertib, an inhibitor of Aurora A kinase, suppresses tumor cell proliferation in liposarcoma cells [91] and was related to favorable clinical outcomes with a high 12-week progression-free rate in 73% of liposarcoma patients [95]. Aurora A and B kinases inhibition by RNAi and pharmacological treatment with a pan Aurora kinase inhibitor AMG 900 reduced tumor proliferation and cell survival in liposarcoma cell lines [96]. MLN8237, an Aurora A kinase inhibitor, showed G2/M phase cell cycle arrest and a cytotoxic effect leading to apoptosis of tumor cells with a significant synergistic effect with chemotherapy agents against LPS, except for cisplatin [97].

## 8. Epigenetic Modification in Liposarcoma

The skin is the most outer layer organ and is exposed to various environmental stimuli. These environmental stimuli provoke epigenetic changes, which regulate the biological modification of DNA without changing the sequences and subsequently influence the degree of gene expression [98]. The importance of epigenetic modification has been observed in various cutaneous skin malignancies, such as squamous cell carcinoma [99], melanoma [100], and lymphomas [101].

Recent studies have identified epigenetic modification in liposarcoma tumor cells. *p16INK4a* gene promoter methylation is observed in 50% of dedifferentiated liposarcomas, not in well-differentiated liposarcomas [102]. *HDAC1* (Histone deacetylase 1) somatic mutations are observed in 8.3% of patients with dedifferentiated liposarcoma [103]. CEBPA (CCAAT/enhancer binding protein, alpha) methylation is seen in 24% of dedifferentiated liposarcomas. Demethylating agent treatment improves the expression of CEBPA in dedifferentiated liposarcoma cells and plays a role in antitumor development. In soft tissue sarcomas, hypoxia is related to a worse prognosis. HIF-2α enhances transcriptional responses under hypoxia and activates soft tissue sarcoma metastasis. An HDAC inhibitor, vorinostat, inhibits tumor growth mediated by *HIF-2α* deletion [104].

The degree of histone H3 at Lys9 trimethylation (H3K9me3) is elevated in dedifferentiated liposarcoma [105]. Kruppel-like factor 6 (KLF6) was decreased in dedifferentiated liposarcoma with increased *H3K9me3* hypermethylation. H3K9me3 inhibition reduced tumor proliferation and increased expression of the adipogenesis regulation factors CEBP (CCAAT/enhancer binding protein) α, CEBPβ, and PPARγ, indicating that enhanced H3K9me3 mediates dedifferentiated liposarcoma-associated dedifferentiation. The overexpression of KLF6 partially induced phenotypic changes in dedifferentiated liposarcoma cells that contributed to the development of adipocytic differentiation.

## 9. Discussion

### 9.1. Usefulness in the Current Treatment

The clinical characteristics of superficial-type ALT, such as tumor size, location, and texture, will be helpful for the early detection of ALT in clinical aspects in order to distinguish from ordinal lipoma. In histology, MDM2 and CDK4 examinations in FISH are a golden-standard method; however, immunostaining for MDM2 and CDK4 might be useful for the diagnosis as an alternative method.

### 9.2. Limitation in the Current Treatment

The current novel treatment options for advanced-form ALT have developed the clinical outcome of ALT; however, they cannot obtain a satisfactory level in clinical outcomes. Although more detailed clinical trial information is necessary, the immunotherapy targeted PD1/PD-L1 pathway may make a breakthrough against the current limitation of treatment for advanced-form ALT—the same as the other malignancies. In addition, other options will be desired to improve the clinical outcome in an advanced form of ALT.

## 10. Conclusions

We summarized a recent update on the characteristics and studies of ALT. There are several studies focused on a possible favorable clinical behavior in superficial-type ALT; however, the actual clinical course, the response to current treatment, and the pathogenesis of superficial-type and deep-type ALT remain unclear. It is a golden-standard method to detect *MDM2* and *CDK4* by FISH for the diagnosis of ALT; however, immunostaining as an alternative method has been investigated. Since technique quality and antibody accuracy are increasing, improvements in immunostaining methods for the diagnosis of ALT can be expected. Recent studies also identified key molecules to regulate the development of ALT as the targeted treatment; however, they still do not reach the satisfactory level to obtain a clinical outcome. Along with the treatment update, it may be realistic to proceed with radical surgical resection as much as possible with early detection and early treatment under the current situation. Therefore, the novel risk factors for the diagnosis of ALT are desired to be discovered in further investigations. In addition, further development of a treatment is required for improvement in the clinical outcome of ALT in the future.

## Figures and Tables

**Table 1:** The positive frequencies of MDM2 and CDK4 in benign lipoma.

Author	MDM2				CDK4			
	Number		Percentage		Number		Percentage	
	Total	Positive	Positivity	Specificity	Total	Positive	Positivity	Specificity
Italiano A, et al. [48]	8	0	0%	100%	8	0	0%	100%
Pilotti S, et al. [49]	19	0	0%	100%	19	0	0%	100%
Dei Tos AP, et al. [50]	18	0	0%	100%	18	2	11.1%	88.9%
Binh MB, et al. [51]	48	2	4.2%	95.8%	44	1	2.3%	97.7%
Sirvent N, et al. [52]	16	1	6.3%	93.7%	16	0	0%	100%
Thway K, et al. [45]	58	10	17.2%	82.8%	58	18	31.0%	69.0%
Clay MR, et al. [38]	96	1	1.0%	99.0%	96	8	8.3%	91.7%
Total	263	14	5.3%	94.7%	259	29	11.2%	88.8%

**Table 2:** The overview of clinical characteristics of cases with superficial-type atypical lipomatous tumor (ALT) in the past literature works.

Case	Author	Age	Sex	Location	Size (cm)	Subjective Symptom	MRI (Septal Structure)	MDM2	CDK4	Treatment	Observation Period (Month)	Recurrence
1	Zámecník [7]	61	Female	Right chest	3 *	N.D	N.D	N.D	N.D	N.D	72	None
2	Ikegami [8]	51	Male	Left back	46 × 52	None	N.D	N.D	N.D	ER	40	None
3	Iwao [9]	75	Male	Back neck	4 *	None	N.D	N.D	N.D	ER	12	None
4	Mathew [10]	26	Male	Right thigh	3 × 3 × 3	N.D	N.D	N.D	N.D	ER	N.D	N.D
5	Tanoue [11]	55	Male	Right back	10 × 9 × 3.5	N.D	+	N.D	N.D	ER	6	None
6	Miyakura [12]	56	Female	Left thigh	11 × 7	N.D	+	+	N.D	MR	N.D	None
7	Satoh [13]	58	Female	Chest	4 *	N.D	-	N.D	N.D	ER	18	None
8	Paredes [14]	56	Female	Abdomen	1 *	N.D	N.D	N.D	N.D	MR	7	None
9		69	Male	Buttock	5 *	N.D	N.D	+	N.D	MR	18	None
10	Suzuki [15]	76	Male	Right thigh	21 × 15 × 5	Pressure	+	N.D	N.D	ER	13	None
11	Tsukamoto [16]	85	Male	Right thigh	15	None	+	N.D	N.D	MR	10	None
12	Kaczmarczyk [17]	57	Female	Left cheek	3 × 2	N.D	-	+	N.D	MR	60	None
13	Sakahara [18]	75	Male	Back neck	35 × 15 × 15	None	+	N.D	N.D	ER	12	None
14	Haneda [19]	43	Female	Back neck	3 × 2	None	-	N.D	N.D	ER	18	None
15	Kurohama [20]	80′s	Male	Left axilla	20	N.D	N.D	+	+	MR	4	None
16	Ogawa [22]	48	Female	Left lower leg	12 × 8 × 5	N.D	N.D	+	+	ER	9	None

* Diameter; ER, extended resection; MR, marginal resection; N.D, not described.
